# Intrinsic Apoptosis Pathway in Fallopian Tube Epithelial Cells Induced by Cladribine

**DOI:** 10.1155/2014/928036

**Published:** 2014-11-05

**Authors:** Ewelina Wawryk-Gawda, Patrycja Chylińska-Wrzos, Marta Lis-Sochocka, Kamila Bulak, Barbara Jodłowska-Jędrych

**Affiliations:** Chair and Department of Histology and Embryology with Experimental Cytology Unit, Medical University of Lublin, Radziwiłłowska 11 Street, 20-080 Lublin, Poland

## Abstract

Cladribine is a purine nucleoside analog which initiates the apoptotic mechanism within cells. Moreover, the available data confirms that cladribine, with the participation of the p53 protein, as well as the proapoptotic proteins from the Bcl-2 family, also induces the activation of the intrinsic apoptosis pathway. However, while there has been a lot of research devoted to the effect of cladribine on lymphatic system cells, little is known about the impact of cladribine on the reproductive system. The aim of our study was to evaluate apoptosis in oviduct epithelial cells sourced from 15 different female rats. In so doing, the sections were stained with caspases 3, 9, and 8. Results suggest that cladribine also induces apoptosis in the oviduct epithelial cells by way of the intrinsic pathway. Indeed, the discontinuing of the administration of cladribine leads to a reduction in the amount of apoptotic cells in the oviduct epithelium.

## 1. Introduction

Cladribine (2-chloro-2′-deoxyadenosine, 2-CdA) belongs to family of purine nucleoside analogs (PNA). This group of drugs was synthesized in the 1970s for the treatment of hematologic diseases. The use of these for this purpose contributed to observations of disturbances within lymphatic cells, in people with a deficiency of deaminase adenosine (ADA). With these people, the excessive accumulation in lymphatic cells of deoxyadenosine-5′-triphosphate (dATP) brought about the destruction of these cells, and an immune deficiency was observed. A similar effect was seen on lymphatic cells incubated along with a synthetic derivative of triphosphate adenosine. Because of the substitution of the hydrogen atom with a chlorine atom in position 2 in the deoxyadenosine ring, cladribine is resistant to ADA action. Cladribine's action is connected with the toxic impact of its main metabolite, 2-chlordeoxyadenosine triphosphate (2-CdATP) on cells. This effect induces their apoptosis.

The cladribine induced apoptotic mechanism has been the subject of intense research, and most of the data obtained confirms the activation of the intrinsic apoptosis pathway by way of the participation of the p53 protein and the proapoptotic proteins from the Bcl-2 family. There has been a lot of research devoted to cladribine action on lymphatic system cells. The results reveal that cladribine permanently lowers CD4^+^ and CD8^+^ lymphocyte levels, as well as B lymphocyte values, and to a smaller degree, the level of NK cells, while the effect on monocytes and neutrophils appears to be of transient character. Apart from the direct impact on the number of lymphocytes, cladribine also acts on the proinflammatory cytokines, inhibiting their secretion [1, 2]. What is more, its suppressive action on lymphatic system cells permits the use of cladribine in treating hematologic malignancies and autoimmune diseases. For the last 30 years, cladribine has been used in the treatment of hairy cell leukemia (HCL)—with good results in most cases. In addition, the positive effects of cladribine in the treatment of chronic lymphocytic leukemia (CLL) have been described [3], as has its efficacy in treating non-Hodgkin lymphoma (NHL) [3], mantle cell lymphoma (MCL) [4], acute myeloid leukemia (AML) [5], myelodysplastic syndromes (MDS) [6], chronic myeloid leukemia (CML) [7], Waldenström macroglobulinemia (WM) [8], histiocytosis, (in children, as well [9]), astrocytomas [10, 11], multiple sclerosis (MS) [2, 12, 13], mast cell disease [14], psoriasis (including psoriatic arthritis [15]), systemic lupus erythematosus (SLE) [16], angioedema, and anaphylaxis [17]. The treatment of younger individuals, even children, with cladribine appears to be a major factor in stimulating a more exact recognition of its actions, due to the possibility of being able to observe long-term reactions [18–21]. By way of these studies, the many side effects induced by cladribine, have been noticed. These are connected mainly with the suppressive effects of 2-CdA on the bone marrow. The consequences of myelosuppression include leukopenia, thrombocytopenia, and anemia. The adverse side effects of cladribine administration also include bacterial, viral, and fungal infections, neuropathy, cardiac insufficiency, nephropathy, disorders of the gastrointestinal tract, musculoskeletal disorders, local skin reactions, teratogenicity, and also secondary malignancies, such as AML, NHL [22–26].

Till now, little has been known about the impact of cladribine on the reproductive system. Therefore, it seems appropriate to study this influence [27]. In our previous studies, we have shown the toxic effect of cladribine action on OSE (ovarian surface epithelium) cells [28]. The oviduct is an important organ of the female reproductive system. The role it plays in reproduction is dependent on the uptake of the oocyte released from the ovaries and, consecutively, its transportation to the uterus. Thus, its action is crucial in providing the proper conditions for fertilization and the survival of the fertilized egg [29]. For performing all the above-mentioned functions, keeping the correct structure and proper functioning of all the elements forming the oviduct is essential, as factors causing structural or functional lesions of the oviduct may lead to infertility [30].

In the present study, we evaluated the impact of cladribine on the oviduct epithelium of healthy rat females at the dose applied in humans for hematologic malignancies. Because numerous studies show that the mechanism of 2-CdA action is based on the induction of apoptosis, in our study we analyzed the expression of effector caspase 3 in the cells of the surface epithelium of the oviduct after cladribine application. Moreover, in order to assess the pathway of apoptosis, we carry out an analysis of caspase 9 expression (which is an intrinsic pathway marker) and caspase 8 (which is a mediator of the extrinsic way of apoptosis). Furthermore to assess the reversibility of the changes caused by cladribine in the epithelial cells we also tested material 4 weeks after 2-CdA therapy.

## 2. Materials and Methods

### 2.1. Experimental Model

White female Wistar rats were used for the study. These were 3- to 4-month-old females with an average body weight of 275 g. Cladribine was applied subcutaneously, interchangeably in the right and left side skin fold at the site of the lumbar spine (the study was approved by the Local Bioethics Commission of Medical University of Lublin-number 126/2001). During the experiment, the animals were housed in cages of a surface area of 0.5 m^2^, with a preserved circadian cycle (12 h/day, 12 h/night), air temperature around 21°C, and relative humidity around 60%. The rats received standard LSM feed and water without limitations. Before starting the experiment, a cytological smear drawn from each female was analyzed to determine the phase of their estrous cycle. In all the studied females, the estrous cycle lasted 4 days and consisted of four phases: proestrus, estrus, metestrus, and diestrus. On the first day of the fourth cycle, drug administration was started.

### 2.2. Research Groups

The animals were randomly placed into 3 groups: one control group (C) and 2 study groups (A and B), each of which consisted of 5 individuals. The animals in the study groups were given the drug at a dose of 0.10 mg/kg BW/24 h, for 7 consecutive days, at exactly the same time. The dose used corresponded to the therapeutic dose used in the treatment of proliferative disorders of the lymphatic system in humans. The control group animals received only food and water without limitations. In order to introduce a stress-inducing factor as that of the study groups, rats in the control group were administered physiological saline subcutaneously in a volume corresponding to the amount of drug administered to rats in the study groups. Decapitation of the animals of both group A and control group was conducted 24 hours after the last injection, that is, on the fourth day of the cycle (the final stage of diestrus). Decapitation of the animals of B study group was conducted 4 weeks after the last injection. Their oviducts were then taken for histomorphological and immunohistochemical tests.

### 2.3. Histological Tests (H&E Staining, Immunohistochemical Tests)

After fixing the material in 10% formalin, the organs were embedded in paraffin blocks. The material was then tailored into 5 micrometers thick sections. The histomorphological evaluation of tissues stained with hematoxylin and eosin (H + E) was performed with the use of a light microscope, at 200x, 400x, and 1000x magnification. Immunohistochemical tests were then done using antibodies directed against caspases 3, 8, and 9 (manufactured by Sigma). The exposure of the antigenic sites was performed thermally by incubation in citrate buffer solution with pH = 6, in a microwave oven at 800 W, for 3 cycles lasting 5 minutes each. To inhibit endogenous peroxidase activity, 0.3% perhydrol (H_2_O_2_) in methanol was used. Normal Serum was also used to block the nonspecific bindings of antigen. The material was incubated in a primary antibody diluted as recommended by the manufacturer (1 : 100) overnight at 4°C. To visualize the reaction, diaminobenzidine (DAB) solution and hematoxylin coloration were used. In the negative control, experiments were conducted in a similar manner, but omitting the specific primary antibody. The material was evaluated with the use of light microscope at 200x and 400x magnification. The percentage of cells with positive expression was calculated in the 300 cells of 3 randomly selected microscopic fields (in magnification of 400x). Moreover, the intensity of expression of individual proteins of fallopian tube epithelial cells was compared. This intensity was graded as low (+), intermediate (++), and high (+++), by using the BX4 image analysis system manufactured by Olympus, with a DP 25 digital camera and the Cell$\hat{\;\;\;}$D software. In addition, the percentage of cells of the given intensity expression in each analyzed field was calculated. The obtained tests results were subjected to statistical analysis with the use of Statistica 10.0 software. The chi-square (*χ* ^2^) test was applied to compare the mean of proteins indicating positive and negative cells in groups. A statistical analysis of the intensity of protein expression in groups was performed with the use of Kruskal-Wallis Test. In this regard, a probability (*P*) value less than 0.05 was considered statistically significant.

## 3. Results

### 3.1. Histomorphological Assessment of the Oviduct Epithelium in H&E Staining

**Figure 1:**
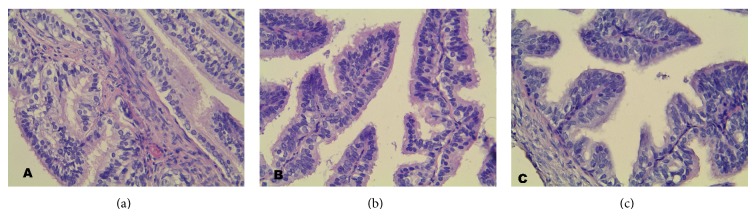
Section of the oviduct epithelium. C: control group, A: study group, B: study group (mag. x400), and H&E staining: The oviduct epithelium showed no discernible pathological changes.

The oviduct epithelium of the analyzed groups of animals, in the histomorphological study, showed no discernible pathological changes under the light microscope at 400x magnification (Figure 1). Moreover, the epithelium lining of the oviducts of the tested females exhibited normal properties of single layer cylindrical epithelium. Moreover in the mucous membrane we observed multiple folds directed along the line of the oviduct. The epithelium was created by two types of cells: ciliated cells and secretory cells, as well as scarce pig cells. We did not observe the supporting cells. The abundant ciliated cells had numerous long cilia on their free surface. The nuclei of the ciliated cells were oval and contained a small amount of heterochromatin and 1 or 2 nucleoli of spherical contour. The cytoplasm of these ciliated cells was poorly eosinophilic. In several single cells, we were able to observe mitotic divisions. Slightly less numerous were the secretory cells. These were characterized by more strongly eosinophilic cytoplasm and by having a considerable quantity of extended intracellular structures. The nuclei of these cells had an oval shape and had 2-3 nucleoli. All cells of the epithelium rested on well-defined basal lamina.

### 3.2. Immunohistochemical Evaluation of Caspase Expressions in Oviduct Epithelial Cells

**Figure 2:**
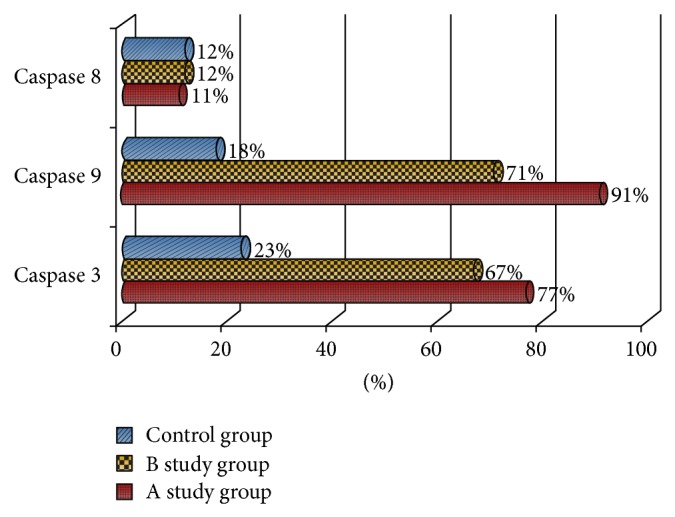
Percentage of positive caspases expression in cells of oviduct epithelium. Positive expression of caspase 8 was rare in all three groups. Positive immunoprecipitation of caspases 3 and 9 was observed more frequently in the study groups than in the control group.

The positive expression of caspases 3 and 9 was seen to be significantly different among the analyzed groups (*P* < 0.05 in the *χ* ^2^ test). In this respect, positive immunoprecipitation of caspases 3 and 9 was observed more frequently in the study groups than in the control group (Figure 2). However, positive expression of caspase 8 was rare in all three groups (*P* > 0.05 in the *χ* ^2^ test).

#### 3.2.1. Expression of Caspase 3 (Figure 3)

**Figure 3:**
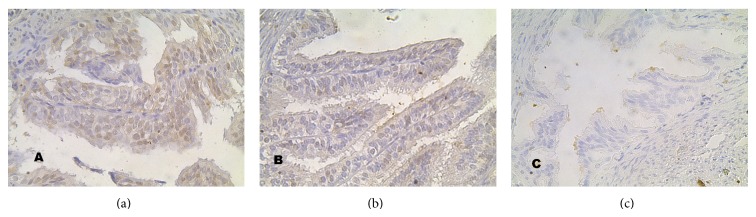
Expression of caspase 3 in oviduct epithelial cells of the control group (C), as well as study groups A and B (mag. x400). The highest intensity expression in group A, the lowest in the control group.

**Figure 4:**
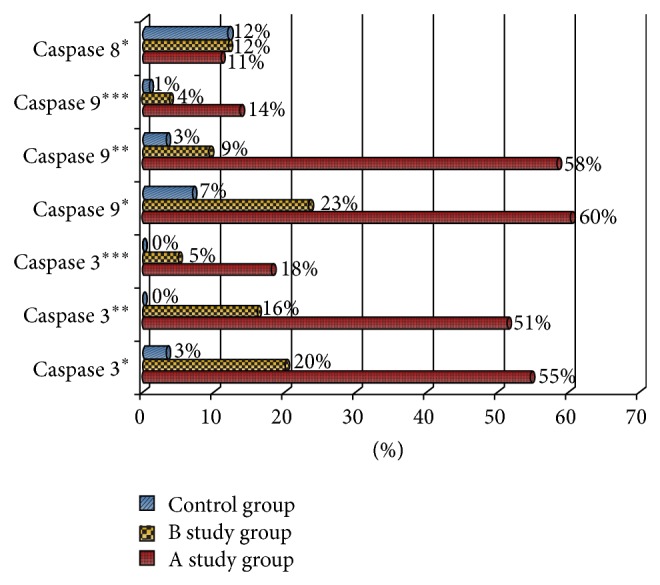
Intensity of caspase expression in particular research group. The expression of caspase 8 was low (^*^) in all groups. The expression of caspases 3 and 9 was at the higher (intermediate (^**^) and high (^***^)) level in the study groups than in the control group.

Regarding the expression of caspase 3 (Figures 3 and 4) in the oviduct epithelial cells of the control group, we observed just low expression. In group B, however, positive caspase expression occurred much more frequently and an intermediate intensity of this expression was observed in 16% of all cells (Figure 4). We did not observe a high intensity of caspase 3 expression in the B group; rather, the highest intensity expression of this was observed in group A, with the animals decapitated 24 hours after last cladribine injection.

#### 3.2.2. Expression of Caspase 9 (Figure 5)

**Figure 5:**
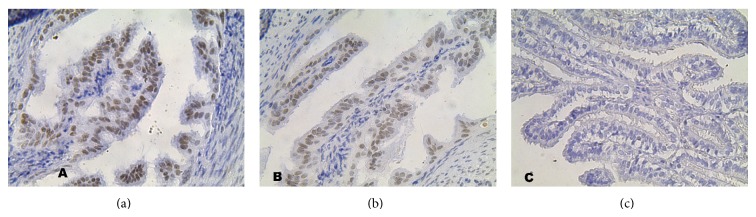
Expression of caspase 9 in the oviduct epithelial cells of the control group C and study groups A and B (mag. x400). The expression of caspase 9 was more intensive in the study groups than in the control group.

A high intensity of caspase 9 expression (Figure 5) was observed mainly in the study groups. This expression was most frequently observed in study group A, more rarely in group B, and the rarest in the control group (Figure 5). However, a low intensity of caspase 9 expression was observed in the 60% of cells of the oviduct of the animals of group A (Figure 4). Moreover, in this group, intermediate intensity was observed in 1/2 of cells, and high intensity in around 14% of cells. High intensity was observed more rarely in group B and only in 1% of cells in the control group.

#### 3.2.3. Expression of Caspase 8 (Figure 6)

**Figure 6:**
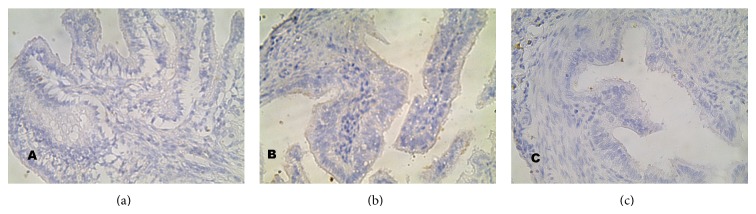
Expression of caspase 8 was at the low level in oviduct epithelial cells of control group (C), as well as study groups A and B (mag. x400).

In the material taken from the animals of all the examined groups, a similar quantity of cells was observed that displayed positive expression of caspase 8 (Figure 6). Both in the material obtained from animals subjected to the action of cladribine and in the tissues of animals given only a saline solution, a minute percentage of positive immunoprecipitates was evident within the cells of the oviduct epithelium. In addition, the intensity of their colour staining was low (Figure 4). A stronger expression was not observed with regard to caspase 8, in the epithelium of the oviducts of any group of animals. Both among the experimental groups and the control group and between the individual experimental groups, a characteristic difference in the appearance of the positive immunohistochemical reaction was not observed. Indeed, in all three analyzed visual fields, the rate of the essential relationship in the *χ* ^2^ test amounted to *P* > 0.05.

### 3.3. Caspase 9 Expression versus Caspase 8 Expression

In the studied material, we observed positive immunoprecipitates of mainly caspase 9, while positive expression of caspase 8 appeared more rarely (Figure 2). In our work, we demonstrated a statistically significant difference between the appearances of the positive expression of caspase 9 and caspase 8 in the cells of the epithelium of the oviduct of animals subjected to the action of cladribine. In contrast, the difference in the degree of caspase precipitation in the epithelium of oviducts of control group's animals (Table 1) was not statistically significant. Moreover, the intensity of the expression of caspase 9 was much higher in the examined tissues of study groups A and B, compared with the expression of caspase 8 (*P* < 0.05 in the Kruskal-Wallis Test, Figure 4).

## 4. Discussion

Cladribine induces the apoptosis of cells, and it penetrates into the interior of the cell through the cellular membrane by way of nucleoside transporters (NT). This is the first phase of the intracorporeal effects of this medicine. Within the cell, cladribine is converted into the active 2-CdATP metabolite [31, 32]. Its presence in the cell leads to a series of enzymatic and structural changes which upset the balance between damaging factors and repair mechanisms. This results in the end of the cell's life. Cladribine works both on actively proliferating and on nonactively proliferating cells, but the cytotoxic effect is multidirectional.

With dividing cells, the 2-CdA action is connected mainly with the inhibition of the deoxynucleotide synthesis that plays a major part in the replication and repair of DNA. Apart from this, 2-CdA suppresses the initiation of ribonucleotide reductase (RR), which is the enzyme that conditions the speed of the synthesis of diphosphodeoxyribonucleotides (dNDP). The limiting of dNDP quantity upsets the balance in the nucleoside triphosphate pool (dNTP), which in turn, is involved in activating endonucleases. This brings about the fracturing of one or both threads of DNA [31, 33]. This effect is additionally strengthened through the inhibition of repair of damaged DNA as a result of the inactivation through cladribine of enzymes such as $\hat{\text{I}}$, polymerase, or DNA polymerases [31, 32, 34, 35].

Apart from this, synthetic nucleosides generated by way of cladribine can join in the polynucleotide chain during the replication of DNA, and this can litigate this process or form double strand breaks, (DSB). The presence of damage within the DNA contributes to the activation of the p53 protein which initiates the repair mechanisms of the cell. However, when damage is too extensive and their repair is not possible, the p53 protein directs the cell towards apoptosis [31, 36]. In cases of the lack of sufficient p53 protein, cladribine can directly activate the Apaf-1 factor or lead to an increased permeability of the mitochondrial membrane to cytochrome c, by way of the independent caspases mechanism. The intracellular calcium ion concentration also probably plays a role in the induction of apoptosis through cladribine [37, 38].

A few studies indicate the presence of an extrinsic pathway for apoptosis, engendered by way of the administration of cladribine. A membrane activation of death receptors FAS/CD95 through 2-CdA was shown by Nomura et al., in a line of human leukaemic MOLT-4 cells [39]. Other possible means of terminating the cell through 2-CdA is through the activation of the MAPK p38 line (p38 mitogen-activated protein kinase) or by way of ERK1/2 (extracellular signal-regulated kinases 1 and 2) [37, 40].

In our study, we assessed the effect produced by cladribine on the cells of the epithelium of the oviducts. We also ascertained the standard pathway of the activation of the apoptosis through the participation of caspases. Moreover, by way of immunohistochemical staining, we were able to determine the expression of the caspase 3 effector which is responsible for the destructive changes in the cell and, therefore, is regarded as being a molecular marker for apoptosis. Initiating caspase 9 plays a role in the intrinsic pathway of the apoptosis, but in the extrinsic pathway the key role is played by the death receptor and by caspase 8 [41–43]. Therefore, determining this expression of those initiating caspases allowed us to determine the pathway for the induction of the apoptosis through cladribine, in the cells of the examined tissues.

Although the epithelium of the oviducts that we analysed is characterized by a typical morphological construction for the epithelium in the diestrus phase, still, in the immunohistochemical examination, differences in the expression of the proteins of the apoptosis line were discernible among the individual research groups. In our examination, the most intense expression of caspase 3 was observed in the cells of the epithelium of the oviducts of animals belonging to experimental group A. This reveals the inductive action of cladribine within the process of the programmed death of the examined epithelium cells. What is more, a difference in intensity of the expression of caspase 3 was shown in our examination to be statistically significant among individual experimental groups, as well as among experimental groups and the control group.

A similar effect was repeatedly observed to have been exerted on unaffected and leukemic cells in the examinations of material obtained from the tissues of affected and control patients, as well as through cell cultures lines [32, 37, 42, 44]. In addition, an increase in the activity of caspase 3 was shown in the majority of the research accompanying the effective treatment by way of cladribine administration, of hyperplastic illnesses. Moreover, Ceruti et al. demonstrated the increase in the activity of caspase 3 in astrocytoma cells subjected to cladribine action [11]. Only in the research undertaken by Galmarini et al. was an increase of the expression of caspase 3 in cells incubated in the environment of cladribine not observed, in spite of the appearance of the death of the cells by way of their dependency on the active p53 protein [45].

Furthermore, in our work, undifferentiated stem cells, also a known target of cladribine action, were found in the epithelium of the oviducts. This was earlier described in a few studies [46–48]. Chow, in his study that was devoted to the impact of cytostatics on cells in the different stage of maturity, showed that cladribine induces the apoptosis of progenitor CD34^+^/CD38^+^ cells [46]. However, Lech-Maranda et al. observed a growth inhibition of granulocyte-macrophage progenitor cells (CFU-GM) and Petzer et al. revealed the same in T-lymphocyte colony forming cells (CFU-TL) subjected to action of cladribine [47, 48]. What is more, previous research showed that 2-CdA was suppressing the maturation of the precursors of erythrocytes and granulocytes, and its action is less intensive towards undifferentiated stem cells, as compared with more directed progenitors of blood cells [46–48].

For the purpose of the verification of the pathway that induces apoptosis through cladribine, we conducted an analysis of the expression of caspase 9 and caspase 8. The results of these analyses confirmed previous data resulting from the majority of conducted examinations relating the activation of apoptosis by cladribine in the intrinsic pathway. In our work, the expression of caspase 9 in the cells of oviduct epithelium of animals belonging to the experimental group and similarly the expression of caspase 3 was much higher when compared with the remaining groups. In addition, the positive expression of caspase 9 was also found in the examined epithelium of the organs of decapitated animals, four weeks following the administration of the last dose of cladribine (group B); however, its intensity was much lower. In our work, the expression of caspase 8, being a marker for the receptor pathway of apoptosis in the studied cells of the epithelium, appeared in a minor percentage of cells and, in fact, was very low. Furthermore, we did not observe a statistical difference between the appearance and intensity of the one caspase in the individual research groups. It is thus possible to conclude that the supply of cladribine did not affect the expression of caspase 8 in the studied material.

In our study we also prove that after 4 weeks of break (7 estrous cycles) in the 2-CdA therapy, the intracellular changes are still present in the epithelium of the oviduct, which is not sufficient time to plan the pregnancy.

Our research also confirmed the earlier studies in which the increase in the activity of caspase 9 was effected by way of the influence of cladribine [41, 42]. However, an increase in the expression was not observed for caspase 8. This was similar to that mentioned by Nomura et al. [39]. The observation of a lower intensity of the expression of caspases 3 and 9 in cells of the taken tissues, 4 weeks following the last injection, confirms prior reports pointing at the passing cytotoxic effect of cladribine on the organism's healthy cells [40, 49, 50].

Current examinations concerning the action of cladribine were conducted on lymphatic cells, and little information is given as to 2-CdA action on the reproductive system. One incidence involving one patient was described. This was an individual, who, 10 months following treatment with cladribine for hairy cell leukaemia, ended her self-administration of oral contraceptives. Next, she became pregnant and gave birth to a healthy child. This indicated that her reproductive function was retained in spite of the administration of cytostatics in the past [51].

The data regarding the influence of analogues of purine nucleosides on reproduction functions results undoubtedly from the epidemiology of hairy cell leukaemia (HCL). Long-term observation of cladribine action is mainly conducted amongst men and women in the postmenopausal age, since this is the group of individuals mostly affected by HCL. At present, however, cladribine is also applied in other diseases—such as in multiple sclerosis, the myelodysplastic syndrome, or in histiocytosis. These are recognized as affecting young people, not to mention children; therefore, knowledge of cladribine's influence on fertility seems to be essential [2, 5, 9, 12, 27].

The changes that we observed in the cells of the epithelium of the oviducts can have a significant effect on the correct functioning of the reproductive system. However, this examination requires confirmation within human studies. Presumably, it is believed that cladribine can weaken the reproduction abilities of women and men and act teratogenically; therefore, it should not be administered to pregnant woman, or to women who are planning pregnancy [27, 52]. Wubah et al. showed in a few examinations conducted on animal models that the teratogenic action of cladribine is connected with the increase in the activity of the p53 protein within the cells of the foetus [53, 54]. However, Lau et al. state that the damaging action on the foetus depended on the kind of species accepting the cladribine [52].

Demonstrating the toxic effect of the given compound on the fertility is not straight forward, on account of the abundance of factors influencing its pathogenesis. Indeed, observing the menstruation regularity within patients, or the presence of Graafian follicles, does not testify to their ability to have an offspring. Moreover, examinations of the semen sometimes do not also deliver enough data [55, 56]. However, ultrasound examinations allow researchers and doctors to evaluate the structure of generative organs and detect possible pathological changes in reproduction abilities which could be restored by way of surgery [57, 58]. Moreover, simultaneous application of various diagnostic methods, along with the control of the hormone economy, can help in the assessment of the impact of drugs for restoring the fertility of treated patients.

## 5. Conclusions

1. Exposing healthy organisms to the action of cladribine does not bring about discernible structural changes in the epithelium of the oviducts visible in the light microscope.
2. Cladribine leads to the induction of apoptosis of the oviducts epithelial cells in the intrinsic pathway.
3. Changes produced by cladribine in fallopian tube epithelial cells are transient, but, after 4 weeks of break (7 estrous cycles) in the 2-CdA therapy, the intracellular changes are still present in the epithelium of the oviduct.
4. Obtained findings require confirmation by way of examinations on human individuals in order to employ the generated data as potential clinical targets.

## Figures and Tables

**Table 1:** The differences in caspase 9 and caspase 8 expression in the oviduct epithelium—a probability (*P*) value in chi-square (χ^2^) test.

	Caspase 9 expression versus caspase 8 expression in A study group	Caspase 9 expression versus caspase 8 expression in B study group	Caspase 9 expression versus caspase 8 expression in control group
chi-square (χ^2^) test	*P* < 0.0001	*P* < 0.0001	*P* = 0.0530
